# Cryptic diversity in Black rats *Rattus rattus* of the Galápagos Islands, Ecuador

**DOI:** 10.1002/ece3.2033

**Published:** 2016-05-05

**Authors:** Sandi Willows‐Munro, Robert C. Dowler, Michael R. Jarcho, Reese B. Phillips, Howard L. Snell, Tammy R. Wilbert, Cody W. Edwards

**Affiliations:** ^1^ School of Life Sciences University of KwaZulu‐Natal PO Box X01 Scottsville 3209 South Africa; ^2^ Department of Biology Angelo State University San Angelo Texas; ^3^ Neuroscience Program Loras College Dubuque Iowa; ^4^ U.S. Fish and Wildlife Service Pacific Islands Fish and Wildlife Office 300 Ala Moana Blvd Rm 3‐122 Honolulu Hawaii; ^5^ Department of Biology and Museum of Southwestern Biology University of New Mexico Albuquerque New Mexico; ^6^ Smithsonian Conservation Biology Institute National Zoological Park Washington District of Columbia; ^7^ Department of Biology George Mason University Fairfax Virginia 22030

**Keywords:** Biological invasions, conservation biology, Galápagos, genetic diversity, invasive species, island biology, phylogeography, rodent

## Abstract

Human activity has facilitated the introduction of a number of alien mammal species to the Galápagos Archipelago. Understanding the phylogeographic history and population genetics of invasive species on the Archipelago is an important step in predicting future spread and designing effective management strategies. In this study, we describe the invasion pathway of *Rattus rattus* across the Galápagos using microsatellite data, coupled with historical knowledge. Microsatellite genotypes were generated for 581 *R. rattus* sampled from 15 islands in the archipelago. The genetic data suggest that there are at least three genetic lineages of *R. rattus* present on the Galápagos Islands. The spatial distributions of these lineages correspond to the main centers of human settlement in the archipelago. There was limited admixture among these three lineages, and these finding coupled with low rates of gene flow among island populations suggests that interisland movement of *R. rattus* is rare. The low migration among islands recorded for the species will have a positive impact on future eradication efforts.

## Introduction

Alien invasive species represent an ever‐growing threat to many global ecological systems. Members of the genus *Rattus* (Fisher de Waltheim) are major vertebrate invaders and have been introduced to vast areas outside their native ranges due to their small size, adaptability, and commensal association with humans (Aplin et al. 2011; Bonhomme et al. 2011). In particular, the Black Rat (*Rattus rattus*) is among the world's worst invasive species (Lowe et al. 2000). The effects of these invasive rodents on endemic biodiversity can be extremely detrimental (Jones et al. 2008; Garba et al. 2014). Native insular species are particularly sensitive to competition and predation from introduced species (Clavero and García‐Berthou 2005), and invasive rodents can also transmit diseases to native animals, resulting in the devastation of indigenous communities (Harris 2009). The 129 volcanic islands and islets of the Galápagos archipelago (Snell et al. 1996) remained undiscovered by humans until 1535. In the time that humans have settled the islands, hundreds of alien species have been introduced. The impacts of these alien species on the native biota of the Galápagos have been widely discussed in the literature (Schofield 1989; Steadman et al. 1991; Desender et al. 1999; Tye 2006; Phillips et al. 2012). Three invasive rodents have colonized the islands in the archipelago: the Black rat (*R. rattus*), Norway rat (*R. norvegicus*), and the House mouse (*Mus musculus*). From historical records and the detailed record of human activities on the islands, the sequence of introductions of these three species to the Galápagos Islands can be hypothesized. The sources of the initial colonizers and exact routes of dispersal across the islands for each species, however, remain to be clarified.

**Figure 1:**
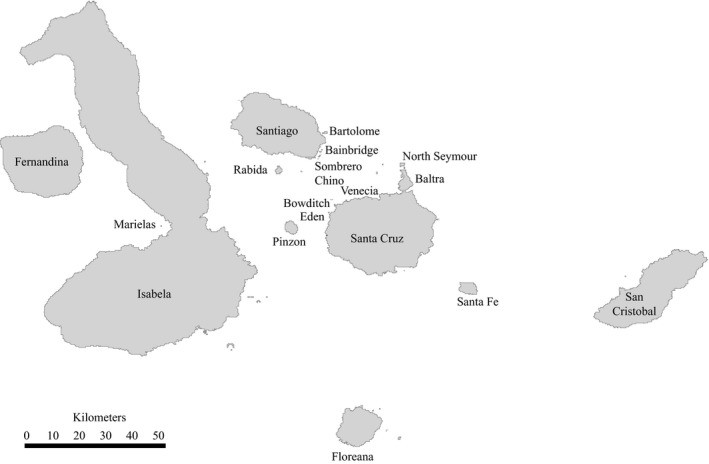
Map of the Galápagos Archipelago indicating the islands where the introduced rodent *Rattus rattus* were sampled.

Of the three alien rodent species, *R. rattus* has invaded the largest number of islands (36) in the archipelago and the species now occurs in a wide range of human and nonhuman associated habitats (Key and Muñoz 1994; Phillips et al. 2012). *Rattus rattus* is believed to have been first introduced to the islands (Fig. 1) between 1684 and 1700 by English buccaneers who established a transient camp near James Bay, Isla Santiago (Patton et al. 1975). Darwin documented the presence of *R. rattus* at Sullivan Bay, Isla Santiago in 1835, from there the species spread to neighboring Isla Bartolomé (Patton et al. 1975). There was no mention of rats on the other islands visited by Darwin, namely Isla San Cristóbal, Isla Floreana, and Isla Isabela. A second introduction of *R. rattus* probably occurred during a second major wave of human activity, with the establishment of a colony on Isla Floreana in 1832 (Patton et al. 1975). The islands of San Cristóbal (1835 and 1869) and Isabela (1893) were both settled by colonists from the Floreana community. Movement of goods among these newly established colonies facilitated the dispersal of *R. rattus* to Isla San Cristóbal and Isla Isabela with the first specimens of this species from these islands collected between 1891 and 1899. A third more recent introduction of *R. rattus* probably occurred during the 1930s on the islands of Santa Cruz and Baltra when these islands were used as a U.S. Army Air Corps base during World War II (Patton et al. 1975). The origin of *R. rattus* populations on Isla Pinzón is unknown. Although whalers used this island during the early‐to‐mid 1800s no permanent human settlement has ever been established there (Patton et al. 1975). Specimens assigned to this species were first recorded from Isla Pinzón as early as 1891, but it is unclear whether Isla Pinzón's *R. rattus* population represents a fourth invasion to the archipelago or whether the *R. rattus* on this island was established as a result of interisland colonization.

Morphological differentiation of island populations of *R. rattus* in terms of body size, shape, and pelage were first reported in 1904 (Heller 1904). The genetic and morphological divergence among *R. rattus* populations across the islands of the archipelago was first empirically tested by Patton et al. in 1975. Based on allozyme frequency, cranial, and mensural characteristics, Galápagos Island *R. rattus* populations were clustered into three main groups: Isla Santiago–Isla Bartolomé group, Isla Floreana–Isla Isabela–Isla San Cristóbal (+Isla Pinzón) group, and Isla Santa Cruz–Isla Baltra group. The Isla Pinzón population was morphologically very distinct from all other island populations and was aligned with the Isla Floreana–Isla Isabela–Isla San Cristóbal group only by the allozyme data (Patton et al. 1975). This latter study broadly supported the colonization pattern suggested by historical records of human activities on the islands and suggested that the high levels of differentiation observed among the three *R. rattus* groups were the result of at least three independent introductions from different source populations. This study also suggested that the morphological and genetic differentiation observed was the result of limited or no gene flow among the different island groups (Patton et al. 1975).

The evolution of taxa native to the Galápagos Archipelago has received much recent attention (Parent et al. 2008; Steinfartz et al. 2009; Štefka et al. 2011; Garrick et al. 2014). Few molecular studies have focused on the invasive species found on the islands (Dudaniec et al. 2010), with the routes used by invasive taxa traditionally reconstructed from historical observational data. Quantifying the genetic variability of invading populations is an important component in estimating the capacity of invaders to respond to new environments and expand from initial point of invasion (Lee 2002; Lavergne and Molofsky 2007). Understanding the invasion pathways and interisland movement of alien rodent species is important for managing and eradicating these taxa from the archipelago, as groups of islands with high rates of migration need to be considered as eradication units (Robertson and Gemmell 2004; Abdelkrim et al. 2007). Rodents have been successfully eradicated from several islets in the Galápagos (DIISE Partners, 2014), and it is essential to understand the potential for their reinvasion. In this study, we determine the genetic structure of the invasive rodent *R. rattus*, on the Galápagos Islands. We aim to provide new insights into the origins (single or multiple introductions) and dispersal patterns of this species across the Galápagos Islands.

## Methods

The Galápagos Archipelago (1°40′N–1°36′S, 89°16′–92°01′W) located off the west coast of Ecuador in the eastern Pacific Ocean is recognized as a World Heritage Site and a biodiversity hotspot (Myers et al. 2000). The islands in the archipelago differ markedly in size. For example, the largest of the islands, Isla Isabela, measures 4588.12 km^2^, (making up half of the total land area of the Galápagos), while several islands like Isla Sombrero Chino are much smaller (0.21 km^2^; Snell et al. 1996). We sampled individuals (*n* = 581) from multiple localities on larger islands and single trapping areas on small islands. Nonetheless, in this study, we refer to all individuals sampled from a particular island as members of the same population. Because smaller islands are often spatially associated with larger islands and as such would be expected to have elevated interisland rates of migrations, smaller landmasses were assigned to larger islands (referred to as proximate regions; Table 1) following Snell et al. (1996).

**Table 1:** Characteristics of the Galápagos Islands included and the number of individuals used in the present study. Islands were assigned to proximate regions following Snell et al. (1996). Coordinates are given as the position of the middle of each island

Island	Proximate region	Position	# individuals
Isla Isabela	Isabela	0°25′30″S, 91°7′0″W	138
Islote Marielas	Isabela	0°35′31″S, 91°5′19.5″W	5
Isla Pinzón	Pinzón	0°36′30″S, 90°39′57″W	77
Isla Santiago	Santiago	0°15′30″S, 90°43′30″W	42
Isla Sombrero Chino	Santiago	0°22′2.5″S, 90°34′55″W	25
Roca Bainbridge	Santiago	0°21′24″S, 90°33′48″W	60
Isla Bartolomé	Santiago	0°16′51″S, 90°32′48″W	15
Isla Santa Cruz	Santa Cruz	0°37′0″S, 90°21′0″W	56
Islote Venecia	Santa Cruz	0°31′10.6″S, 90°28′29.1″W	18
Islote Punta Bowditch	Santa Cruz	0°31′57.7″S, 90°31′1.7″W	11
Isla Eden	Santa Cruz	0°33′41.0″S, 90°32′11.2″W	21
Isla Baltra	Santa Cruz	0°25′30″S, 90°16′30″W	26
Isla Seymour Norte	Santa Cruz	0°23′30″S, 90°17′0″W	18
Isla Floreana	Floreana	1°17′0″S, 90°26′0″W	53
Isla San Cristóbal	San Cristóbal	0°48′30″S, 89°25′0″W	16

### Material collection

Fieldwork was conducted from 1995 through 2008 with sampling from 15 islands in the archipelago. *Rattus rattus* were collected primarily in Tomahawk live traps but were also sampled with Sherman live traps. Voucher specimens of all species have been deposited in the Angelo State Natural History Collections, Angelo State University (San Angelo, TX, USA). Permits were provided by the Parque Nacional Galápagos. Field methods followed guidelines for use of wild mammals in research (Gannon and Sikes 2007).

### Microsatellite typing

DNA was extracted using DNeasy tissue kits (Qiagen, Valencia, CA), and DNA was stored in elution buffer at −20°C. Successful extraction was verified by visualization on a 1% agarose gel, and extracts were diluted 1:10 for subsequent amplifications. Ten loci were amplified in *R. rattus* (D11Mgh5, D16Rat81, D5Rat83, D9Rat13, D7Rat13, D10Rat20, RfgC2, RfgD6, RfgL1, RfgL4) using published primers (Jacob et al. 1995; Hinten et al. 1999). The locus D7Rat13 was monomorphic and was not included in subsequent analyses of this species. In each case, the forward primer was labeled with a fluorescent dye (6‐FAM; Invitrogen Carlsbad, California, United States). Each PCR contained 2 *μ*L of diluted template DNA (~50 ng DNA), 0.1 *μ*L AmpliTaq^®^ DNA polymerase Applied Biosystems, Foster City, California, United States, 2.0 *μ*L 10× reaction buffer, 1.5 *μ*L of 8 mmol/L dNTPs mixture, 1.0 *μ*L of each 10 *μ*mol/L primer, 1.0 *μ*L betaine, and 11.4 *μ*L DEPC H_2_O for a final reaction volume of 20 *μ*L. Positive and negative controls were used during PCR to ensure accuracy of results within and across PCRs and microsatellite size calls. Following optimization, the thermal profile consisted of the following parameters: one cycle at 95°C for 5 min, followed by 35 cycles at 95°C for 20 sec, locus‐specific annealing temperature for 30 sec, 72°C for 90 sec with an increase of 5 sec/cycle, and a final extension of 72°C for 10 min. Amplified fragments were diluted as necessary and run with ILS 600 size standard (Promega Madisin, Wisconsin, United States) in HiDi formamide on an SCE 9610 (SpectruMedix, LLC). Chromatograms were analyzed using GenoSpectrum software (SpectruMedix, LLC) with both automated peak detection and manual inspection. Allele size data were exported from GenoSpectrum and then binned and scored using a custom PERL script as well as manual correction in Microsoft Excel.

### Data analysis

#### Genetic variation

FreeNA (Chapuis and Estoup 2007) was used to estimate null allele frequencies (for each locus and island population) following the expectation maximization algorithm of Dempster et al. (1977). Departure from Hardy–Weinberg expectations was tested with exact tests in GENEPOP 3.4 (Rousset 2008) with a Bonferroni correction for multiple tests. Standard measures of genetic diversity were calculated in GenAIEx 6.4 (Peakall and Smouse 2006) and *F*_STAT_ 2.9 (Goudet 2001) and include average observed heterozygosity (*H*_O_), average expected heterozygosity (*H*_E_), allelic richness (*A*_R_), and the fixation index (*F*).

#### Population structure

Genetic differentiation among island populations of *R. rattus* were examined in Arlequin 3 (Excoffier et al. 2005) using pairwise *F*_ST_ estimates with 10,000 random permutations of the data and Bonferroni correction to assess significance. Pairwise *F*_ST_ values between populations were also calculated after applying the excluding null alleles (ENA) correction, implemented in FreeNA. This method corrects for the bias introduced by the presence of null alleles (Chapuis and Estoup 2007). The 95% confidence intervals for corrected pairwise F_ST_ values were obtained by bootstrapping 1000 times over loci. The uncorrected pairwise F_ST_ matrix was then visualized using principal coordinate analysis (PCoA) in GenAIEx. Isolation by distance was tested using Mantel tests; the pairwise geographic distance matrix (in km) was calculated using the latitude and longitude coordinates of the center of each island (Snell et al. 1996). The significance of the correlation between genetic diversity (*F*_ST_) measures and geographic distance was assessed with 10,000 random permutations in GenAIEx. The hierarchical genetic differentiation (among regions, among populations, and within populations) was statistically examined using an analysis of molecular variance (AMOVA) in GenAIEx.

Bayesian clustering analysis performed in STRUCTURE version 2.2 (Pritchard et al. 2000; Falush et al. 2003) was used to estimate the number of genetic clusters represented by the assayed genotypes without the use of prior population assignment information. Ten independent runs were conducted in STRUCTURE with the number of proposed clusters (*K*) ranging from 1 to 10. Each run was performed under the admixture model with correlated allelic frequencies, using 100,000 Markov chain Monte Carlo iterations and a burn‐in of 10,000 iterations, which was sufficient for likelihood stabilization. The optimal number of clusters was assessed using the Evanno et al. (2005) method as implemented in STRUCTURE harvester (Earl 2009).

A neighbor‐joining (NJ) tree representing the relationships among sampling localities was constructed using the PHYLIP package v. 3.69 (Felsenstein 2005). The subprogram SEQBOOT was used to generate 1000 bootstrap replicates before the calculation of the distance matrices using GENEDIST. Cavalli‐Sforza's and Edwards chord distances (Cavalli‐Sforza and Edwards 1967) were used to measure genetic distance as this metric has been suggested to provide the greatest likelihood of phylogenetic accuracy when using microsatellite data (Takezaki and Nei 1996). The subprograms NEIGHBOUR, CONSENSE, and DRAWTREE were all used in the NJ topology construction. To further clarify the sequence of interisland colonization events, the NJ tree was rooted using the Isla Santiago population as the root of the tree.

A network based on the shared allele distance (Chakraborty and Jin 1993) among individuals was built with EDEN_ETWORKS_ (Kivelä et al. 2015). The effective percolation threshold (Dpe) was calculated automatically and is the point at which the network will fragment into smaller subnetworks and is calculated by considering the maximum distance among individuals in the network. Only links with values smaller than or equal to the percolation distance (in this case 0.56) were used to construct networks. The structure observed in the network drawn using the estimated percolation distances can be considered to represent the first major level of genetic structure present in the data. Decreasing the percolation threshold would elucidate shallower population structure.

#### Demographic history

The relative contribution of genetic drift versus migration on the population structure of this invasive rodent species in the Galápagos was tested using a Bayesian approach implemented in the program 2MOD version 0.2 (Ciofi et al. 1999). Simulations were used to compare the likelihood of two models (migration‐drift equilibrium vs. drift alone) for explaining the genealogy of alleles among island populations (Ciofi et al. 1999). The migration‐drift model assumes that the allele frequencies in the sampled populations are the result of a balance between gene flow and genetic drift under an island model of migration. The drift alone model assumes that the population structure observed among sampled populations is as a result of drift alone (Ciofi et al. 1999). Two independent simulations of 3 × 10^6^ iterations were performed with the first 10% of the iterations which were discarded as burn‐in. Convergence of MCMC chains was assessed using Tracer v1.5 (Rambaut and Drummond 2007) by evaluating effective sample size (ESS) values. Stationarity was confirmed when ESS values exceeded 200 for all parameters. The relative contribution of migration and drift on each island grouping was examined using the parameter *F*. This value is the probability that two genes share a common ancestor within a population. Larger *F* values would suggest that a population is strongly influenced by genetic drift, while *F* values close to zero indicate that migration is also playing a role in the demographic history of the population. Posterior distributions of *F* were visualized in Tracer.

Contemporary migration rates were estimated using BayesAss 3.0.1 (Wilson and Rannala 2003). Five independent runs each consisting of 3 × 10^6^ iterations were performed (Faubet et al. 2007). A burn‐in value of 10^6^ was used, and delta values for all parameters were adjusted such that 20–60% of the total changes were accepted. Stationarity was again assessed in Tracer using ESS values (Rambaut and Drummond 2007).

## Results

### Population genetics and summary statistics

The mean null allele frequencies for five loci (D11Mgh5, D5Rat83, D16Rat81, D10Rat20, and RfgL1; data not shown) exceeded 10%. The presence of null alleles in data can be problematic for calculations of genetic variability as they can decrease within‐population variance and so inflate F_ST_ values. Population differentiation across all populations of *R. rattus* was high, however, even after implementing the ENA correction (*F*_ST_ = 0.27, CI: 0.25–0.30, *P *<* *0.0001; after null allele correction, *F*_ST_ = 0.27, CI: 0.25–0.29), indicating substantial population subdivision across the islands. Original pairwise *F*_ST_ estimates (uncorrected) all fall within the 95% CI of the ENA‐corrected estimates (data not shown). Thus, the effects of any null alleles present in the *R. rattus* data set are likely minimal and all further analysis was performed using data from all loci.

High genetic diversity was observed within the Galápagos *R. rattus* population, reflected in the high allelic richness (which corrects for the biasing effects of sample size variation) estimated (*A*_R_ mean = 3.247; Table 2). Populations of *R. rattus* were also characterized by a large number of loci not conforming to Hardy–Weinberg equilibrium, with all populations except one (Islote Marielas; Table 2) deviating significantly from that expected if the population were in equilibrium.

**Table 2:** Summary of genetic variability of populations of *Rattus rattus* on the Galápagos

Island	*N*_A_	*H*_O_	*H*_E_	*A*_r_	*F*	HWD
Floreana	0.136 (0.152)	0.281 (0.091)	0.44 5 (0.081)	2.273 (0.825)	0.353 (0.185)	D11Mgh5, D5Rat83, D16Rat81, D10Rat20, RfgC2
Baltra	0.066 (0.068)	0.383 (0.091)	0.458 (0.098)	2.489 (1.047)	0.153 (0.088)	D5Rat83, D16Rat81, RfgL1
Bartolome	0.092 (0.128)	0.445 (0.096)	0.533 (0.043)	2.406 (0.601)	0.215 (0.161)	D5Rat83, RfgL1
Bainbridge	0.173 (0.121)	0.122 (0.048)	0.339 (0.069)	1.856 (0.576)	0.584 (0.150)	D11Mgh5, D5Rat83, D16Rat81, D10Rat20, D9Rat13, RfgL1, RfgL4
Bowditches	0.115 (0.128)	0.212 (0.064)	0.348 (0.07)	1.974 (0.66)	0.344 (0.157)	D11Mgh5, D5Rat83, D16Rat81
Eden	0.046 (0.111)	0.280 (0.099)	0.333 (0.095)	1.86 (0.795)	0.119 (0.134)	D16Rat81, RfgL1
Isabela	0.134 (0.068)	0.314 (0.057)	0.508 (0.089)	2.53 (0.967)	0.384 (0.087)	D11Mgh5, D5Rat83, D16Rat81, D10Rat20, D9Rat13, RfgL1, RfgD6, RfgL4
Marielas	0.013 (0.037)	0.344 (0.096)	0.368 (0.09)	1.885 (0.824)	0.063 (0.162)	None
Pinzon	0.125 (0.127)	0.191 (0.075)	0.348 (0.073)	1.886 (0.572)	0.459 (0.153)	D11Mgh5, D5Rat83, D16Rat81, D10Rat20, RfgL1, RfgL4
Sombrero Chino	0.123 (0.122)	0.355 (0.095)	0.524 (0.081)	2.616 (0.902)	0.350 (0.135)	D11Mgh5, D5Rat83, D16Rat81, D10Rat20, D9Rat13, RfgL1, RfgL4
San Cristobal	0.152 (0.094)	0.338 (0.071)	0.581 (0.081)	2.919 (0.957)	0.438 (0.072)	D11Mgh5, D16Rat81, D10Rat20, D9Rat13, RfgL1, RfgL4
Santa Cruz	0.129 (0.096)	0.295 (0.075)	0.467 (0.086)	2.368 (0.768)	0.353 (0.098)	D11Mgh5, D5Rat83, D16Rat81, D10Rat20, D9Rat13, RfgL1, RfgD6, RfgL4
North Seymour	0.081 (0.106)	0.358 (0.086)	0.479 (0.099)	2.525 (0.99)	0.195 (0.101)	D5Rat83, D16Rat81, D10Rat20, RfgL1
Santiago	0.129 (0.122)	0.288 (0.075)	0.477 (0.09)	2.492 (0.965)	0.364 (0.122)	D11Mgh5, D5Rat83, D16Rat81, D10Rat20, RfgL1
Venecia	0.114 (0.129)	0.231 (0.089)	0.380 (0.096)	2.081 (0.877)	0.497 (0.153)	D11Mgh5, D5Rat83, D16Rat81, RfgL1, RfgL4
Total	0.108 (0.113)	0.296 (0.021)	0.439 (0.022)	3.247 (1.05)	0.328 (0.035)	All loci, all populations *P* < 0.001

Standard error in parentheses.

*N*_A,_ mean null allele frequency; *H*_O_, mean observed heterozygosity; *H*_E_, mean expected heterozygosity, *A*_r_, mean allelic richness, *F*, fixation index; HWD, loci exhibiting Hardy–Weinberg disequilibrium.

As significant genetic differentiation among island populations was found, we expected more recently colonized islands to have less genetic variation as a result of population bottlenecks during introduction. This pattern however was not recovered in spatial genetic structure of *R. rattus* across the Galápagos. For example, the genetic diversity of *R. rattus* population in Isla Santiago (*H*_E_ = 0.0.477, *A*_R_ = 2.492; Table 2) and Isla Santa Cruz (*H*_E_ = 0.467, *A*_R_ = 2.368) is comparable although historical records suggest that these islands were colonized at least 200 years apart. There was also no evidence for an increase in genetic diversity with increasing size of island, with many smaller islands having populations that contain allelic richness comparable with populations sampled from larger landmasses – for example; although the number of *R. rattus* collected from Isla Isabela and Isla Sombrero Chino differ, these two islands have comparable levels of observed and expected heterozygosities and allelic richness despite a considerable difference in island size (Snell et al. 1996). The interpretation of this pattern may be tenuous as geographically proximate islands may operate as single units.

### Population structuring

Pairwise *F*_ST_ values (Table 3) were all highly significant in comparisons among *Rattus* populations. Pairwise *F*_ST_ values > 0.1 were observed in the majority of population comparisons (82% of comparisons, Table 3). Even populations sampled from islands belonging to the same proximate region (Table 1) and only separated by a few kilometers typically exhibited *F*_ST_ > 0.05. For example, Isla Santiago – Roca Bainbridge, *F*_ST_ = 0.066. Mantel tests suggest that this high genetic diversity observed was not due to the phenomenon of isolation by distance (*r* = 0.063, *P* = 0.304). The AMOVA revealed that most of the genetic variance could be explained by within population differences (58%; Table 4). Substantial fixation indices were, however, also recovered from among region comparisons (*F*_RT_ = 0.342, *P* = 0.01).

**Table 3:** Pairwise *F*_ST_ values between populations of *Rattus rattus*: Isla Floreana (FLO), Isla Baltra (BAL), Isla Bartolome (BAR), Roca Bainbridge (BNB), Islote Punte Bowditches (BOW), Isla Eden (EDN), Isla Isabel (ISB), Isolte Marielas (MAR), Isla Pinzon (PNZ), Isla Sombrero Chino (SOM), Isla San Cristobal (SCB), Isla Santa Cruz (SCZ), Isla Seymour Norte (SEY), Isla Santiago (SAN), Islote Venecia (VEN). All *F*_ST_ values were significant

*Rattus rattus*	FLO	BAL	BAR	BNB	BOW	EDN	ISB	MAR	PNZ	SOM	SCB	SCZ	SEY	SAN	VEN
FLO	0.000														
BAL	0.303	0.000													
BAR	0.247	0.166	0.000												
BNB	0.392	0.222	0.121	0.000											
BOW	0.371	0.072	0.226	0.283	0.000										
EDN	0.351	0.074	0.221	0.297	0.110	0.000									
ISB	0.214	0.236	0.160	0.282	0.267	0.243	0.000								
MAR	0.268	0.333	0.242	0.303	0.395	0.393	0.135	0.000							
PNZ	0.298	0.206	0.150	0.230	0.282	0.263	0.198	0.370	0.000						
SOM	0.260	0.160	0.059	0.072	0.220	0.237	0.159	0.216	0.184	0.000					
SCB	0.203	0.163	0.125	0.194	0.198	0.209	0.104	0.218	0.144	0.105	0.000				
SCZ	0.293	0.039	0.158	0.214	0.055	0.036	0.187	0.286	0.169	0.146	0.120	0.000			
SEY	0.311	0.038	0.158	0.195	0.058	0.071	0.199	0.330	0.191	0.149	0.154	0.021	0.000		
SAN	0.299	0.162	0.077	0.066	0.225	0.234	0.195	0.264	0.198	0.020	0.122	0.146	0.150	0.000	
VEN	0.345	0.050	0.201	0.250	0.047	0.080	0.244	0.392	0.233	0.205	0.180	0.028	0.037	0.202	0.000

**Table 4:** Differentiation among proximate region and island populations determined using analysis of molecular variance (AMOVA)

Source	Variance explained (%)	*P*‐value	Fixation index
*Rattus rattus*			
Among regions	34	0.01	*F*_RT_ = 0.342
Among populations	8	0.01	*F*_SR_ = 0.119
Within populations	58	0.01	*F*_ST_ = 0.421

**Figure 2:**
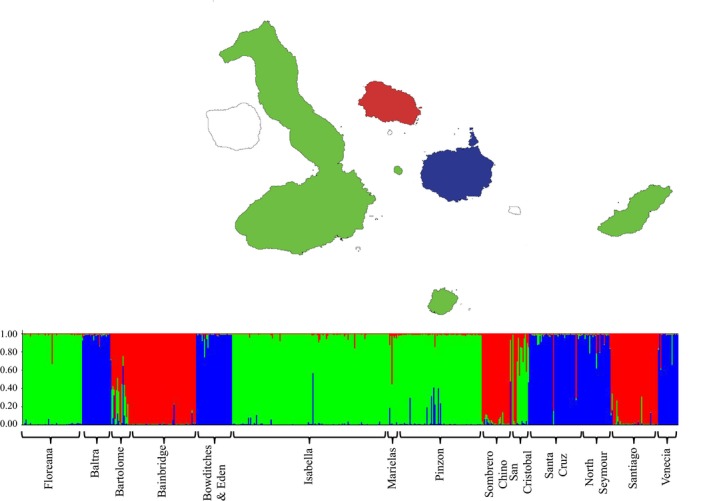
Estimated population structure for *Rattus rattus* on Galápagos from STRUCTURE analysis (*K* = 3). In the STRUCTURE graphs, each individual is represented by a thin vertical line, which is partitioned into K colored segments that represent the individual's estimated membership fractions. Individuals were sorted according to island of collection. The distribution of genetic clusters is shown on the map, with islands colored according to the STRUCTURE results.

**Figure 3:**
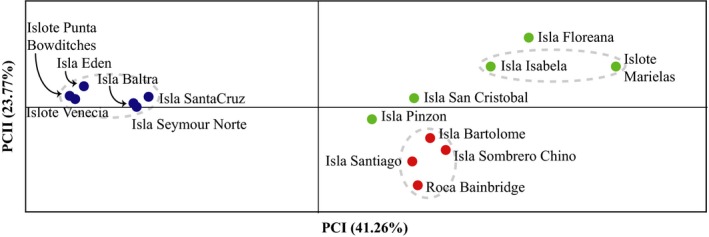
Principal coordinates analysis (PCoA) calculated using pairwise *F*_ST_ estimates between populations of *Rattus rattus*. The first three axes explained 79.13% of variation. Island populations were colored according to the STRUCTURE cluster to which the majority of individuals on the island could be assigned (see Fig. 2). Stippled lines include islands assigned to the same proximate region (see Table 1).

Genetic clustering analyses indicated the presence of multiple unique evolutionary clusters of invasive rodent on the Galápagos Islands. The results of the PCoA and Bayesian STRUCTURE clustering were broadly similar. The most likely number of genetic clusters (*K*) identified in STRUCTURE analyses using the Evanno et al. (2005) method was three (Fig. 2). STRUCTURE and PCoA clustered populations according to proximate regions, such that islands geographically closer to each other tend to be closely related genetically (Figs. 2, 3). This trend was evident for *R. rattus* populations belonging to the islands in close proximity to Isla Santa Cruz, namely Isla Seymour Norte, Isla Baltra, Islote Venecia, Isla Eden, and Islote Punte Bowditches, which clustered as a unit in the PCoA plot (Fig. 3).

**Figure 4:**
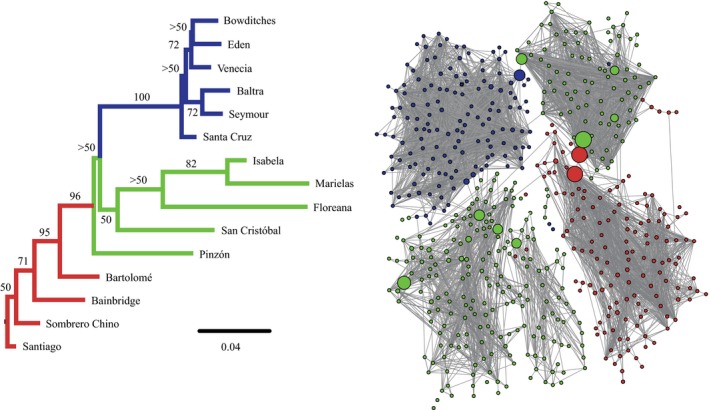
Neighbor‐joining trees (left) and networks (right) of *Rattus rattus* on the Galápagos. The NJ topology based on microsatellite allele frequencies in populations is rooted using the island population, suggested by historical records, as the first site of introduction to the archipelago. The distance matrix is based on Cavalli‐Sforza's chord distance and was bootstrapped 1000 times. The network topology is based on shared alleles’ distances. Only links with distances equal to or smaller than the percolation thresholds (0.56) are shown. Genotypes are color coded based on clusters suggested by STRUCTURE.

The genetic subdivision and sequence of colonization as suggested by historical records (Key and Muñoz 1994) is generally congruent with the NJ topology with some notable exceptions (Fig. 4). Island populations associated with the initial Isla Santiago colonization (Isla Santiago, Isla Sombrero Chino, Roca Bainbridge, and Isla Bartolomé) were surprisingly not recovered as monophyletic. The second hypothesized introduction suggested to have centered around Isla Floreana and including the islands of Islote Marielas, Isla Isabela, and Isla San Cristóbal (excluding Isla Pinzon) was recovered as monophyletic, but only with weak branch support (50% bootstrap support; Fig. 4). The third suggested introduction including Isla Santa Cruz, Isla Seymour Norte, Isla Baltra, Islote Venecia, Isla Eden, and Islote Punta Bowditch formed a well‐supported clade (100% bootstrap support; Fig. 4). In contrast to the findings of Patton et al. (1975) based on allozymes and the Bayesian STRUCTURE analysis of the microsatellite data in this study, the NJ analysis of *R. rattus* does not support the placement of the *R. rattus* population on Isla Pinzón in close association with the Isla Floreana introduction; instead, the Isla Pinzón island population is placed sister to a weakly supported clade including the Isla Floreana and Isla Santa Cruz lineages.

The network analysis supports the results of the NJ and STRUCTURE analysis. In the *R. rattus* network constructed using percolation threshold (Dpe = 0.56; Fig. 4), individuals collected on the islands associated with Santiago and Santa Cruz formed two distinct clusters. A cluster including specimens collected mainly from Pinzón clustered separately from populations on the islands of Isabela, Marielas, Floreane, and San Cristóbal. This Pinzón cluster is epiphyletic as it also includes genotypes sampled on Isabela and San Cristóbal.

### Gene flow and demography

**Figure 5:**
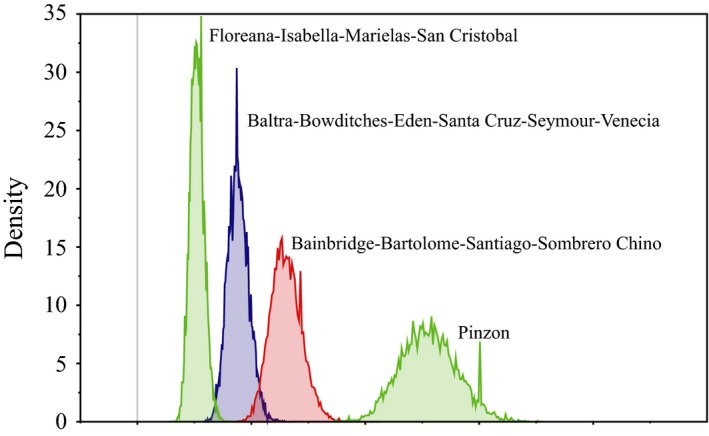
The marginal posterior density for *F* is plotted for the populations of *Rattus rattus* on the Galápagos. Island populations were assigned to clusters as suggested by STRUCTURE. Analysis was run with Isla Pinzón separated from the Isla Floreana–Islote Marielas–Isla Isabela–Isla San Cristóbal cluster.

The migration‐drift model was favored by 2MOD, both when individuals were assigned to islands (*P* = 1.0) and when island populations are assigned to the clusters suggested by the STRUCTURE analysis (*P* = 1.0). This suggests that the genetic structure among the island populations of this invasive rodent species results from a combination of migration and genetic drift. There was, however, considerable variation in the contribution of gene flow and genetic drift among island populations of *R. rattus* (Fig. 5). The Isla Pinzón population had much higher *F* values than those of the other island populations (mean *F* value = 0.512; 95% HPD = 0.42, 0.61), suggesting that genetic drift has played a much greater role in the demographic history of this population (Fig. 5). There was almost no change in *F* value of the Isla Floreana–Islote Marielas–Isla Isabela–Isla San Cristóbal cluster when the Isla Pinzón *R. rattus* population is added to this grouping (mean *F* value = 0.102; 95% HPD = 0.08, 0.12, result not shown).

Multiple runs of BayesAss yielded consistently low estimates (<1% and confidence intervals that overlapped zero) of contemporary gene flow indicating limited migration among most of the island populations of *R. rattus* (results not shown). Migration rates exceeding 1% were observed between the *R. rattus* population of Isla Santa Cruz and populations on the geographically proximate islands of Isla Seymour Norte (0.20, 95% CI: 0.054), Isla Baltra (0.22, 95% CI: 0.05), Islote Venecia (0.20, 95% CI: 0.056), Isla Eden (0.21, 95% CI: 0.051), and Islote Punta Bowditch (0.16, 95% CI: 0.066). Similarly, migration rates higher than 1% were observed among the Santiago *R. rattus* population and populations on Isla Sombrero Chino (0.22, 95% CI: 0.05), Isla San Cristóbal (0.08, 95% CI: 0.018), and Isla Bartolomé (0.18, 95% CI: 0.059). Low migration rates with 95% CI with lower bounds including zero were also observed when *R. rattus* individuals were assigned to clusters suggested by STRUCTURE analysis.

## Discussion

We demonstrate high levels of genetic variability at microsatellite loci in populations of the invasive rodent *R. rattus* present on the islands. The levels of heterozygosity for the *R. rattus* across the Galápagos (*R. rattus*:* H*_O_ = 0.12–0.45) are within the lower range observed in other archipelago populations (e.g., Guadeloupe islands, *H*_O_ = 0.43–0.68, Abdelkrim et al. 2005; Sainte Anne islets, *H*_O_ = 0.31–0.58, Abdelkrim et al. 2007; Larezzi Mediterranean archipelago, *H*_O_ = 0.31–0.71, Abdelkrim et al. 2009; U.S. Virgin Islands, *H*_O_ = 0.04–0.88, Savidge et al. 2012). Low levels of genetic diversity have been observed in some invasive species, as founder populations often represent a subset of the species‐wide gene pool (Frankham 2005). The genetic diversity observed in *R. rattus* on the Galápagos Islands is the genetic signature of a recent bottleneck and propagule pressure.

Geographically isolated island populations are expected to have less genetic variation than continental populations due to factors such as founder effects, genetic drift, and reduced migration. This is indeed the case in the *Rattus* species in the Galápagos, which showed lower observed heterozygosity than that reported in studies of continental populations (USA, *H*_O_ = 0.41–0.7, Lack et al. 2013) and populations on other larger islands (New Zealand, *H*_O_ = 0.73–0.78, King et al. 2011; *H*_O_ = 0.79–0.81, Abdelkrim et al. 2010).

Results from the present study support findings from earlier work that suggest multiple introductions of *R. rattus* occurred in the archipelago (Patton et al. 1975). The geographic distributions of alleles within the species allow some general conclusions to be made. We did not find a hierarchical distribution of diversity in relation to island size. That is, larger islands did not exhibit elevated levels of genetic diversity. This is in contrast to what has been found in other insular rodent populations (e.g., Calmet et al. 2001). One explanation for our results is that smaller islands are generally found adjacent to larger islands. We explored this possibility and expected the geographic location (proximate identity) to have increased the number of potential colonization events leading to populations on smaller islands being representative of the genetic diversity available on neighboring larger landmasses. Our analyses did not support this hypothesis as islands only separated by a few kilometers typically showed significant genetic differentiation and low levels of contemporary migration. The genetic diversity observed on smaller islands could be the result of rapid local adaptation, with individuals on smaller islands becoming quickly isolated, both ecologically and genetically. The limited admixture of genetic lineages present on islands as inferred by the STRUCTURE analyses could suggest that once an island has been colonized by a particular genetic lineage, the invasive rodent populations on the island are in some way resilient to reinvasions. This pattern of genetic partitioning is particularly surprising on Isla Santa Cruz, which has the largest settled human population in the archipelago. Because it represents the urban center of the Galápagos, we expected populations of *R. rattus* sampled from this island to have the genetic signatures of all three of the genetic lineages recorded from across the archipelago, but surprisingly limited genetic admixture was noted on Isla Santa Cruz. This finding was corroborated by our analysis showing little gene flow was evident. The resilience of island populations of invasive rodents to re‐invasion has also been observed in *M. musculus* on the sub‐Antarctic Kerguelen archipelago (Hardouin et al. 2010). The authors of this study suggest that populations on small islands can become more quickly ecologically and genetically isolated than mice on the mainland. This would make it difficult for newly arriving rodents to invade already established populations. Even if a subsequent invasion took place, the genetic contribution of the small number of new migrants would not significantly alter the genetic profile of the island population.

### Phylogeography of *R. rattus* in the Galápagos

The genetic structuring observed in the microsatellite data for *R. rattus* suggest that the Galápagos Islands were invaded by at least three (if not four) separate colonization events from different source populations. The geographic patterning of the genetic variation for this species is in agreement with the colonization pattern described by Patton et al. (1975). Historical data point to Isla Santiago as the initial site of colonization. Our data also attribute the populations of *R. rattus* on the geographically proximate islands of Isla Sombrero Chino, Rocas Bainbridge, and Isla Bartolomé to this first wave of colonization. A second independent introduction of *R. rattus* to the archipelago likely occurred on Isla Floreana associated with the establishment of Villamil's settlement in 1832. The spread of *R. rattus* individuals belonging to this genetic grouping from the source population to the islands of Isla Isabela and Isla San Cristóbal was likely through human‐mediated dispersal. It is then likely that humans facilitated the dispersal of *R. rattus* to Islote Marielas from Isla Isabela. The most recent introduction of *R. rattus* occurred on the islands of Isla Baltra and Isla Santa Cruz, with individuals belonging to this latter genetic cluster also colonizing the adjacent islands of Isla Seymour Norte, Islote Venecia, Isla Eden, and Islote Punte Bowditch. The distinctiveness of the *R. rattus* on the Isla Pinzón as suggested by Patton et al. (1975) was again highlighted by the present study. STRUCTURE analysis clustered the Isla Pinzón *R. rattus* with populations on Isla Floreana, Islote Marielas, Isla Isabela, and Isla San Cristóbal. The PCoA plots, NJ topology, and percolation network for *R. rattus*, however, did not closely associate the Isla Pinzón population with the Isla Floreana genetic grouping. This genetic differentiation coupled with previously reported morphological distinctiveness of this population (Patton et al. 1975) suggests that the population of *R. rattus* on Isla Pinzón may have been established by a fourth distinct colonization event. Alternatively, members of the Isla Floreana genetic grouping may have initially seeded the population on Isla Pinzón, but genetic drift coupled to an extended period of isolation could be responsible for the genetic differentiation of the *R. rattus* population on this island. Although there are fewer historical records documenting the introduction and spread of the other invasive rodents, *M. musculus* and *R. norvegicus,* across the archipelago, it seems likely that the phylogeographic pattern of these species on the islands has also been shaped by multiple independent colonization events.

The biodiversity on several islands in the archipelago have already benefited from eradication of other invasive mammal species (Carrion et al. 2011). The phylogeographic information provided by this study is important for the proper management and conservation of these unique islands. Our data suggest that the invasion of the Galápagos by rats is strongly linked to human settlement and movement across the archipelago, but that dispersal events by rodents between islands may be rare, based on the low gene flow estimates. In this case, conservation efforts that focus on effective prevention of inadvertent movement of invasive rodents between islands could have large‐scale effects on the preservation of island native biota and will contribute significantly toward the eventual eradication of these invasive rodents from the Galápagos.

## Conflict of Interest

None declared.

## Data Accessibility

Microsatellite genotypes are available for download at http://datadryad.org under DRYAD Repository entry doi: 10.5061/dryad.1s88t
